# Ongoing Remission Nineteen Years after High-dose Chemotherapy for Oligometastatic Breast Cancer; What Can We Learn from this Patient?

**DOI:** 10.7759/cureus.433

**Published:** 2015-12-24

**Authors:** Tessa G Steenbruggen, Sabine C Linn, Sjoerd Rodenhuis, Gabe S Sonke

**Affiliations:** 1 Department of Medical Oncology, Netherlands Cancer Institute, Plesmanlaan 121, 1066 CX Amsterdam, the Netherlands; 2 Department of Molecular Biology, Netherlands Cancer Institute, Plesmanlaan 121, 1066 CX Amsterdam, the Netherlands

**Keywords:** medical oncology, breast cancer, high-dose chemotherapy, brca, oligometastatic

## Abstract

Metastatic breast cancer is generally considered incurable. However, some patients show an exceptional response to treatment and enjoy long-term survival in good health. Here, we present a remarkable example of a patient who is still in remission 19 years after high-dose chemotherapy and locoregional treatment for limited oligometastatic breast cancer. We will outline our rationale for this treatment to explain her excellent response and suggest strategies to select larger patient groups that could similarly benefit from existing treatment approaches.

## Introduction

Treatment for patients with metastatic breast cancer remains a challenge. Only 26% of patients with distant metastasis in the United States survive for more than five years [1]. However, some women with metastatic breast cancer show much better responses to therapy and can possibly be cured. Here, we present a patient with limited oligometastatic breast cancer who responded extremely well to high-dose bifunctional alkylating chemotherapy and radiotherapy.

## Case presentation

**Figure 1:**
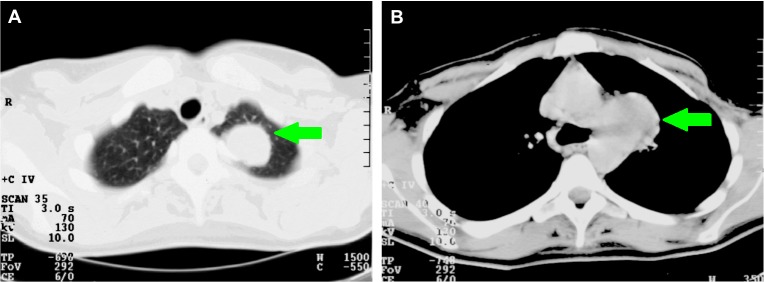
Axial computed tomography (CT) scan in January 1996 pre-treatment. A. The CT scan shows a mass in the left upper lobe of the lung. B. The CT scan shows a large mass in the aortic pulmonary window, reaching from the lateral side of the aortic arch to the left and right pulmonary arteries.

In 1996, a 32-year-old, pre-menopausal woman presented with pain located at the sternum and a hoarse voice due to paralysis of the laryngeal recurrent nerve. A computed tomography (CT) scan of the chest revealed a 6 cm mass in the left upper lobe of the lung (Figure 1A) and enlarged mediastinal lymph nodes on the left side (Figure 1B). Bone scintigraphy showed no bone metastasis. Genetic testing with Sanger sequencing revealed a pathogenic germline *BRCA1* mutation (185delAG on chromosome 17). She had breast cancer of the left breast three years earlier during her second pregnancy. No metastasis was noted at that time, and the breast cancer was treated with mastectomy. Two years later, she underwent prophylactic mastectomy of the right breast. Pathologic evaluation of the primary tumor showed a poorly differentiated invasive ductal carcinoma, negative for the estrogen receptor (ER) and progesterone receptor (PR). Later evaluation also revealed negativity for the human epidermal growth factor receptor 2 (HER2/*neu*). Biopsies of the metastatic sites were unfortunately not possible; however, clinical and radiographic findings showed a recurrence of the breast cancer to be most likely.

**Figure 2:**
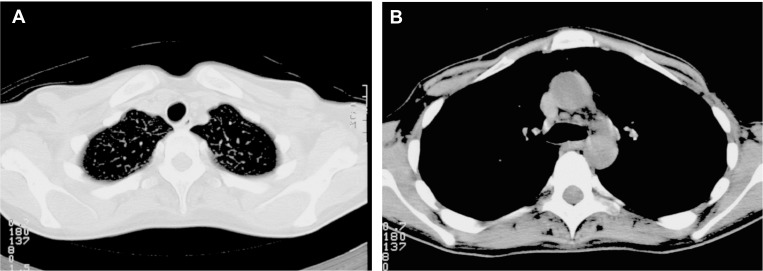
Axial computed tomography (CT) scan in June 1996, two months after high-dose chemotherapy and consolidation radiation A. The CT scan shows no detectable tumor in the lung. B. The CT scan shows no detectable mass in the aortic pulmonary window.

She was enrolled in a clinical trial that aimed to determine the feasibility and efficacy of high-dose chemotherapy with peripheral blood progenitor cell (PBPC) support in women with advanced-stage breast cancer. After two cycles of 5-fluorouracil 500mg/m^2^, epirubicin 120mg/m^2^, and cyclophosphamide 500 mg/m^2^ (FE_120_C), the mass in the lung was much smaller, and her voice improved. She subsequently received three cycles of high-dose chemotherapy consisting of cyclophosphamide 4000mg/m^2^, thiotepa 320 mg/m^2^, and carboplatin 1060 mg/m^2 ^(target AUC 13.3 mg/ml/min) in combination with PBPC support [2]. The high-dose chemotherapy induced a complete radiologic remission of the metastatic sites (Figures 2A-2B). She subsequently received locoregional consolidation radiation to the left apical lung and mediastinum (total dosage: 50 Gray). Due to high-dose chemotherapy, she developed irreversible alopecia and ovarian suppression. Clinical and radiographic evaluations were performed at regular intervals over a period of 19 years and have shown no evidence of disease. She remains in an excellent condition.

## Discussion

### Oligometastatic breast cancer

Approximately five percent of the patients with metastatic breast cancer survive for over ten years. Long-term survival is more often seen in patients with limited metastatic disease, often referred to as ‘oligo’metastatic disease [3]. Oligometastatic breast cancer is variably defined as a metastatic disease with a maximum of one to five metastases beyond the regional lymph nodes. Oligometastatic cancer is assumed to be a disease with limited metastatic potential compared to widespread metastatic cancer and is therefore believed to have the potential to be cured [4]. 

Oligometastatic breast cancer may warrant an aggressive, multidisciplinary approach with curative intent focusing on eradication of the primary tumor, the detected metastases, and circulating micro-metastases [3]. Many studies report on locoregional approaches in oligometastatic disease and invariably find improved outcomes after metastasectomy or high dose radiotherapy [5]. However, as most data stem from non-randomized, phase II studies, it is difficult to draw definite conclusions about the true effect of the interventions. The best results are seen when all metastases are removed, as the source of growth factors and suppression of anti-tumor immunity is reduced [3]. Adding systemic therapy to metastasectomy further improves outcome [6]. We, therefore, considered an intensive, multidisciplinary approach for our patient to improve her chance for long-term survival.

### High-dose alkylating chemotherapy and homologous recombination deficiency

Another important aspect of the treatment our patient received is the high-dose, bifunctional alkylating chemotherapy. High-dose chemotherapy in the treatment of breast cancer patients is controversial, as it has not shown a survival benefit in an unselected group of breast cancer patients [7]. However, in patients with a triple negative tumor, high-dose chemotherapy reduces the risk of death by 33% compared to conventional chemotherapy [7]. These regimens contain potent inducers of DNA double-strand breaks (DSB), which are highly effective in tumors with an inadequate DNA repair system. The tumor suppressor genes *BRCA1* and *BRCA2* are involved in the repair of DNA DSB via homologous recombination. When this mechanism is inactivated, tumor cells rely on less adequate repair mechanisms. This is called homologous recombination deficiency (HRD) and causes genome instability and consequently apoptosis of tumor cells. HRD can be caused by silencing the *BRCA1 *or *BRCA2* function in several ways. Examples involve a germline mutation in either the *BRCA1* or *BRCA2* gene, hypermethylation of the *BRCA1* promotor region, or defects in the Fanconi anemia pathway [8]. Analysis of the DNA copy-number pattern of breast tumors can identify HRD according to a *BRCA1-* or *BRCA2-*like profile [9-10]. We previously showed that patients with high-risk breast cancer and a *BRCA1- *or *BRCA2*-like profile derive important benefit from high-dose, alkylating chemotherapy in comparison to conventional chemotherapy (hazard ratio (HR) for overall survival: 0.19, 95% confidence interval (CI): 0.08 to 0.48) [9]. We think that selecting oligometastatic patients based on HRD for high-dose chemotherapy can improve outcome for more patients. A randomized clinical trial studying this hypothesis is currently open for accrual (NCT01646034).

## Conclusions

The patient presented here with oligometastatic breast cancer, and a mutation in the *BRCA1* gene showed an excellent response to an intensive, multidisciplinary therapeutic approach including high-dose, alkylating chemotherapy and local treatment. From this case, we can learn two key things that are valuable for other patients. First, the excellent response within a metastatic setting may be explained by approaching oligometastatic breast cancer with curative intent. Second, homologous recombination deficiency (for example, due to a mutation in the *BRCA1* or *BRCA2* gene) may make tumors more sensitive to high-dose, bifunctional alkylating chemotherapy. We, therefore, encourage further research in this field to optimally select the best treatment for patients with oligometastatic breast cancer.
